# Novel Metformin-Containing Antibacterial Composite for Root Caries Restorations

**DOI:** 10.3390/ma19142963

**Published:** 2026-07-09

**Authors:** Ayman Altamimi, Ibrahim Ba-Armah, Heba Alqarni, Nader Almutairi, Yazeed Altamimi, Mohammad Alenizy, Abraham Schneider, Jirun Sun, Michael D. Weir, Hockin H. K. Xu

**Affiliations:** 1 Dental Biomedical Sciences PhD Program, Graduate School, University of Maryland, Baltimore, MD 21201, USA; aaltamimi@umaryland.edu (A.A.); halqarni@umaryland.edu (H.A.); nader.almutairi@umaryland.edu (N.A.); yaltamimi@umaryland.edu (Y.A.); 2 Department of Biomaterials and Regenerative Dental Medicine, School of Dentistry, University of Maryland, Baltimore, MD 21201, USA; 3 Department of Restorative Dental Sciences, College of Dentistry, University of Hail, Hail 55475, Saudi Arabia; mh.alenizy@uoh.edu.sa; 4 Department of Restorative Dental Sciences, College of Dentistry, Imam Abdulrahman Bin Faisal University, Dammam 31441, Saudi Arabia; imbaarmah@iau.edu.sa; 5 Department of Pediatric Dentistry and Orthodontic Sciences, College of Dentistry, King Khalid University, Abha 61421, Saudi Arabia; 6 Department of Conservative Dental Sciences, College of Dentistry, Prince Sattam bin Abdulaziz University, Alkharj 11942, Saudi Arabia; 7 College of Dentistry, Ohio State University, Columbus, OH 43210, USA; schneider.1505@osu.edu; 8 ADA Forsyth Institute, Cambridge, MA 02142, USA; jsun@forsyth.org; 9 Center for Stem Cell Biology & Regenerative Medicine, School of Medicine, University of Maryland, Baltimore, MD 21201, USA; 10 Marlene and Stewart Greenebaum Cancer Center, School of Medicine, University of Maryland, Baltimore, MD 21201, USA

**Keywords:** metformin, hPDLSCs, DMAHDM, root caries, periodontal tissue regeneration, stem cells, antibacterial composite

## Abstract

Background: Tooth root caries and periodontal tissue loss remain major challenges in elderly and periodontally compromised patients, while current restorative materials lack combined antibacterial and regenerative properties. Objective: The objective of this study was to develop a novel metformin-containing antibacterial composite for root cavity restorations to prevent recurrent caries and potentially promote periodontal tissue regeneration. Methods: Experimental composites contained 5% dimethylaminohexadecyl methacrylate (DMAHDM), varying metformin concentrations (2.5–15%), and glass fillers. Mechanical and antibacterial properties as well as cytocompatibility toward human periodontal ligament stem cells (hPDLSCs) were investigated. Results: The experimental composites achieved flexural strengths of 67.9 to 50.1 MPa (*n* = 6), significantly higher than (41.3 ± 3.4 MPa) of commercial control Vitremer (*p* < 0.05), while maintaining clinically acceptable elastic moduli of 3.7–3.1 GPa. Experimental composites achieved an 8-log reduction in *Streptococcus mutans* (*S. mutans*) biofilms and significantly reduced lactic acid production and metabolic activity compared to biofilms on commercial controls. The composite containing 15% metformin demonstrated acceptable cytocompatibility toward hPDLSCs under clinically relevant diluted conditions, matching commercial controls (*p* > 0.1). Conclusions: These findings suggest that this novel composite possesses potent antibacterial activity, acceptable cytocompatibility, and adequate mechanical properties, making it promising for multifunctional root caries restorations.

## 1. Introduction

Root caries is caused by biofilm-induced demineralization of cementum and dentin at exposed root surfaces due to the loss of periodontal attachment and gingival recession [1]. The cementum layer of the root is thin and has low levels of minerals, making it very vulnerable to early dissolution by acids; the rapid rate of progress of lesions beneath the dentin layer after the loss of cementum can be attributed to the lower mineral content of dentin when compared to enamel and the higher organic matrix content of dentin [2]. An epidemiologic study conducted using a cross-sectional design within the framework of the National Health and Nutrition Examination Survey (NHANES) from 2017 to 2020 in the United States found that 18.2% of U.S. adults aged 50 years and older were affected by root caries, indicating the high frequency of occurrence of this condition among the aging population [3]. Similarly, the Fourth National Oral Health Survey of China, which took place between 2015 and 2016, found that root caries occurred among 61.9% of Chinese adults who were 65–74 years old, further reinforcing the fact that root caries has a significant global burden [4]. Periodontal loss is one of the causes of root caries because the recession of the gums creates an environment where cariogenic biofilm can come into contact with the root surface [5]. For example, a clinical study found that individuals experiencing gingival recession had significantly higher rates of root caries development than did individuals who experienced no gingival recession [5]. Furthermore, a population-based epidemiologic study conducted in Japan established that gingival recession was strongly correlated with increased rates of root caries development in elderly individuals [6].

One way to resolve the issues of secondary caries includes the creation of antimicrobial dental restorative products that have an inhibitory effect on bacterial development [7]. The use of early antimicrobial approaches included the incorporation of chlorhexidine into dental resins; unfortunately, the antibacterial effects of these materials degraded over time due to material degradation and the release of the agent [8]. Dental composites containing silver nanoparticles demonstrated longer-lasting antibacterial activity, inhibited the formation of biofilm by *Streptococcus mutans* (*S. mutans*), and provided greater antibacterial activity [9]. Quaternary ammonium methacrylates are considered a major improvement over earlier generations of antimicrobial dental restoratives since they provide long-lasting antibacterial activity through a mechanism of killing by direct contact with the microorganisms [10]. Dimethylaminohexadecyl methacrylate (DMAHDM), in particular, has been identified as a compound that provides strong antibacterial activity [11]. Previous work demonstrated that dental composites containing DMAHDM reduced biofilm development of *S. mutans* by 99.9% [11]. This finding was reinforced by another study, which demonstrated that DMAHDM-containing composites retained long-term antibacterial activity without reducing the mechanical strength of the composite [12]. A biomaterials study confirmed that DMAHDM-containing composites significantly reduced biofilm acid production and mineral loss [13].

Metformin is a biguanide medication that was originally derived from the plant Galega officinalis and first synthesized in 1922 prior to its clinical introduction in France in 1957 for the treatment of type 2 diabetes mellitus [14]. Following its approval by the FDA in 1994, metformin has become one of the most widely used medications in the world [15]. In addition to lowering blood sugar, metformin has demonstrated significant regenerative properties in mineralized tissues [16]. Experimental studies have shown that metformin stimulates the differentiation and mineralization of osteoblasts via the activation of AMP-activated protein kinase signaling [16]. An in vitro study showed that metformin stimulated odontogenic differentiation and mineralization of dental pulp stem cells [17]. Additionally, an animal study demonstrated that metformin stimulated alveolar bone regeneration and periodontal wound healing [18]. Metformin has also been shown to exhibit anti-inflammatory effects and stimulate periodontal tissue regeneration [19]. Importantly, metformin has demonstrated strong regenerative effects on human periodontal ligament stem cells (hPDLSCs) [20]. A stem cell study showed that metformin stimulated the proliferation and osteogenic differentiation of hPDLSCs [20]. An in vitro study further demonstrated increased alkaline phosphatase activity and mineral deposition in hPDLSCs treated with metformin [21]. Most recently, a dental biomaterials study demonstrated that metformin-containing dental resin stimulated both osteogenic and cementogenic differentiation of hPDLSCs and stimulated periodontal and tooth root regeneration [22].

Despite these promising data, there is currently no report that has investigated the combination of metformin and DMAHDM in a resin matrix containing UDMA/TEG-DVBE and its multifunctional effects on antibacterial activity, mechanical performance, and cytocompatibility. This gap still exists in the literature concerning restorative materials for root carious lesions where the material with antimicrobial action does not have any regenerative property, or vice versa. To the authors’ knowledge, no previous experimental study has combined DMAHDM and metformin in a UDMA/TEG-DVBE resin-based restorative system for root caries application. The present study aimed to (1) develop and test a new antibacterial dental composite containing metformin as a root cavity filling restoration; (2) investigate how DMAHDM and metformin influence the mechanical, physical, and antibacterial characteristics of this dental composite when it is exposed to *S. mutans*, and (3) determine the potential cytotoxicity of this new dental resin composite toward hPDLSCs. It was hypothesized that the inclusion of metformin and DMAHDM in a UDMA/TEG-DVBE resin would yield a composite with strong antibacterial efficacy to inhibit *S. mutans*, acceptable physical and mechanical properties for root cavity restoration, and good cytocompatibility to support hPDLSCs.

## 2. Materials and Methods

### 2.1. Formulation of Resin Filling with DMAHDM and Metformin

The light-curable resin matrix was prepared by mixing urethane dimethacrylate (UDMA; Esstech, Essington, PA, USA) and triethylene glycol divinylbenzyl ether (TEG-DVBE) at mass fractions of 55.8% and 44.2%, respectively, following previous studies [23]. The photoinitiating system consisted of 0.2% camphorquinone (CQ; Aldrich, Saint Louis, MO, USA) as the photosensitizer and 0.8% ethyl 4-N,N-dimethylaminobenzoate (4EDMAB; Aldrich, Saint Louis, MO, USA) as the accelerator. This resin formulation is referred to as UV resin throughout this study.

The antibacterial monomer DMAHDM was synthesized via a modified Menschutkin reaction following established protocols [24]. The reaction mixture consisted of 10 mmol of 1-bromohexadecane (BHD; TCI America, Portland, OR, USA) and 10 mmol of 2-(dimethylamino) ethyl methacrylate (DMAEMA; Sigma-Aldrich, St. Louis, MO, USA), dissolved in 3 g of ethanol in a 20 mL scintillation vial. The mixture was stirred at 70 °C for 24 h. After evaporation of the solvents, DMAHDM was isolated as a distinctive white, waxy solid. It was then added to UV resin; thus, the resin composite contained 5% DMAHDM by weight.

The resin matrix (UV resin + 5% DMAHDM) made up 30% of the total composite, while the filler comprised the remaining 70%. Silanized barium boroaluminosilicate glass filler particles (1.4 µm; Dentsply Sirona, Milford, DE, USA) were used to mechanically reinforce the resin composite. To investigate the effect of metformin incorporation, seven experimental groups were prepared by progressively replacing portions of the reinforcing fillers with metformin powder, which served as a bioactive co-filler within the composite system. Metformin hydrochloride powder (Sigma-Aldrich, St. Louis, MO, USA) was incorporated at increasing concentrations, with corresponding reductions in reinforcing filler content. The experimental control group (0% metformin) consisted of 70% reinforcing fillers without metformin. In the experimental groups, metformin was incorporated at concentrations of 2.5%, 5%, 7.5%, 10%, 12.5%, and 15%, with reinforcing filler content reduced to 67.5%, 65%, 62.5%, 60%, 57.5%, and 55%, respectively. Two commercially available dental restoration products served as commercial controls in this study; one was a resin composite (Heliomolar HB packable resin composite, Ivoclar Vivadent AG, Schaan, Liechtenstein) and resin-modified glass ionomer cement (Vitremer™, 3M ESPE, St. Paul, MN, USA). The composition of the final experimental and control groups is shown in Table 1.

### 2.2. Mechanical and Physical Properties

#### 2.2.1. Flexural Strength and Elastic Modulus

Resin bars (2 × 2 × 25 mm) were fabricated using a stainless-steel mold for mechanical testing. To prevent the formation of an air-inhibited layer, Mylar strips were applied to the bilateral surfaces during the curing process. Photopolymerization was executed for 60 s per side at an irradiance of 1200 mW/cm^2^ utilizing a Labolight DUO unit (GC, Tokyo, Japan). Following fabrication, the specimens underwent a two-stage conditioning protocol: an initial 24 h dry storage period at 37 °C, followed by immersion in deionized (DI) water for an additional 24 h at 37 °C. Mechanical properties (*n* = 6) were quantified via a three-point bending configuration to determine the flexural strength and elastic modulus. Testing was performed on a Universal Testing Machine (MTS, Insight 1, Cary, NC, USA) equipped with a 10 mm support span and configured to a constant crosshead speed of 1 mm/min [25].

#### 2.2.2. Degree of Polymerization Conversion (DC)

The extent of polymerization of a dental composite was determined using FT-IR spectroscopy (Nicolet 6700, Thermo Fisher Scientific, Waltham, MA, USA). Spectra were collected over the range of 400 to 4000 cm^−1^ at a resolution of 4 cm^−1^ and 32 scans per sample. The extent of the polymerization process was achieved through monitoring the absorbance decrease due to the reaction of the C=C at 1637 cm^−1^ and referencing this value against the absorbance at 1583 cm^−1^, which served as an internal standard. Spectra (*n* = 3) were collected before photopolymerization and after curing for 40 s (Bluephase Style, Ivoclar Vivadent; 1100 mW/cm^2^). The curing conditions for DC measurement (40 s at 1100 mW/cm^2^) differ from those used for mechanical specimen fabrication (60 s at 1200 mW/cm^2^) because DC measurement by FTIR-ATR uses very thin resin films placed in direct contact with an ATR crystal. The short curing time prevents excessive saturation of the sensor, as this could prevent reliable measurements. The conditions used here have been previously reported in other studies as being typical of DC measurement [26,27]. Since the Vitremer control group is a dual-cure material, the DC was determined by utilizing the internal reference peak at 1720 cm^−1^ and took place after 24 h in order to measure the DC based on chemical curing processes that occur in this type of material [28].

#### 2.2.3. Composite Hardness

Hardness was assessed via the Vickers microhardness indentation technique. Bars of resin, sized 2 × 2 × 12 mm for every test group (*n* = 5), were fabricated as described in Section 2.2.1, and stored in distilled water at 37 °C for 24 h before analysis. Microhardness testing was conducted using a Vickers microhardness tester (HMV II; Shimadzu Corporation, Kyoto, Japan). A standardized load of 100 gf (980.7 mN) was applied to the specimen surface with a dwell time of 10 s. The resulting indentation diagonals were evaluated using a 20× objective lens to ensure optimal optical resolution. To ensure statistical reliability, four indentations were made on each specimen, and the Vickers Hardness Number (VHN) was automatically derived by the system based on the diagonal lengths of the indentations, following standard methodology [29].

### 2.3. S. mutans Biofilm Model

#### 2.3.1. Resin Samples for Biofilm Testing

Experimental resin disks (8 mm diameter; 1 mm thickness) were fabricated with a sample size of *n* = 6. Each specimen was subjected to dual-sided photopolymerization for 60 s at an irradiance of 1200 mW/cm^2^ utilizing a Labolight DUO curing unit (GC, Tokyo, Japan). Following a 24 h post-cure incubation at 37 °C, the disks were submerged in distilled water and underwent continuous agitation for 1 h to facilitate the elution of residual, unreacted monomers [30]. Sterilization was subsequently performed via ethylene oxide gas (Anprolene AN 74i, Andersen, Haw River, NC, USA). To ensure the complete elimination of gaseous residues, the sterilized specimens underwent a mandatory seven-day degassing protocol in accordance with the manufacturer’s guidelines [31].

#### 2.3.2. Bacterial Inoculation and Biofilm Formation

The Institutional Review Board at the University of Maryland, Baltimore, approved the use of bacterial strains for this research (HP 00052180). Due to its role as the main bacterium responsible for causing tooth decay, *S. mutans* (UA159) was used to generate the biofilm in these experiments. The bacteria were cultured in brain heart infusion broth (Sigma-Aldrich, St. Louis, MO, USA) for 16–18 h at 37 °C, 5% CO_2_. All cultures were prepared similarly to provide a consistent inoculation dose. A Genesys 10 S Spectrophotometer (Thermo Scientific, Waltham, MA, USA) was utilized to prepare a standardized inoculum of 10^7^ CFU/mL by comparing OD_600_ values vs. previously developed and validated standard curves. After placing each composite resin disk in a 24-well plate containing 1.5 mL of BHI culture medium supplemented with 2% sucrose, the disks were incubated at 37 °C, 5% CO_2_ for 24 h to facilitate initial biofilm attachment. After this period, the composite disks were transferred to fresh 24-well plates containing 1.5 mL of new media with sucrose and incubated for another 24 h to allow mature biofilm development, resulting in a total biofilm formation period of 48 h.

#### 2.3.3. Biofilm Colony-Forming Unit (CFU) Counts

The resin disks (*n* = 6) that contained the biofilm were placed in a 24-well plate with 1 mL of phosphate-buffered saline (PBS). The biofilms were mechanically removed and dispersed via ultrasonication and vortexing. Following the removal, bacterial suspensions generated during the above process were diluted to a range from 10^1^ to 10^6^. Next, the suspensions were plated on BHI agar plates and incubated for 48 h at 37° C in a 5% CO_2_ environment. Post-incubation, the number of colonies was counted under magnification (Reichert Quebec Darkfield Colony Counter, Depew, NY), and CFUs in each biofilm were estimated based upon the number of colonies per dilution factor [32].

#### 2.3.4. Biofilm Metabolic Activity (MTT)

The degree of metabolic viability of biofilms grown on resin specimens was determined using an MTT 3-(4,5-dimethylthiazol-2-yl)-2,5-diphenyltetrazolium bromide colorimetric assay. Established biofilm samples were placed in 24-well culture plates that contained 1 mL of MTT dye (0.5 mg/mL in PBS), which was then incubated at 37 °C in a 5% CO_2_ atmosphere for 1 h. The established biofilm specimens were removed from the initial plates and placed into secondary 24-well plates with 1 mL of dimethyl sulfoxide (DMSO) per well to dissolve the crystals of the resultant formazan product; this process occurred in a light-protected area at room temperature for 20 min. Finally, 200 µL aliquots from the DMSO solution above were pipetted into a 96-well plate and analyzed by spectrophotometry. Absorbance readings at 540 nm were taken using a SpectraMax M5 Microplate Reader (Molecular Devices, Sunnyvale, CA, USA); higher absorbance readings were used to indicate greater levels of metabolic activity of the biofilm [33].

#### 2.3.5. Lactic Acid Production by Biofilms

Following 48 h of biofilm formation on the resin surfaces, the samples were placed in 24-well culture plates containing Buffered Peptone Water (BPW) (Aldrich, St. Louis, MO, USA) with 0.2% sucrose. These samples were then placed into an incubator at 37 °C in a 5% CO_2_ atmosphere for 3 h. Lactate concentrations in the BPW were quantified by the lactate dehydrogenase enzyme, using a microplate reader (SpectraMax M5, Molecular Devices, Sunnyvale, CA, USA) and measuring the absorbance at 340 nm. This procedure was performed in triplicate [34,35].

### 2.4. hPDLSCs Cytotoxicity

Cytotoxicity testing was performed on hPDLSCs under an approved protocol from the University of Maryland (IRB Protocol No. HP-00079029). PDL tissues were obtained from premolars extracted at the University of Maryland dental clinic from individuals aged 12–26 years undergoing orthodontic treatment. hPDLSCs were isolated and characterized following the methods in previous studies [36]. Cells at passage 5 were maintained in fibroblast medium (FM) containing 20% fetal bovine serum (FBS) and 1% penicillin–streptomycin. Once cell viability exceeded 90%, cells were transferred to 96-well plates at a density of 5000 cells per well. Resin disks (8 mm diameter, 1 mm thickness) were prepared, sterilized with ethylene oxide gas, and vacuum degassed for seven days before use. Each disk was immersed in 2 mL of culture medium at 37 °C for 24 h to generate extraction eluents. The total surface area of each disk was calculated as 1.2 cm^2^ (two flat circular faces of 0.5 cm^2^ each and a curved lateral surface of 0.2 cm^2^). Divided by the extraction volume of 2 mL, this yielded a surface area to volume ratio of 0.6 cm^2^/mL, following ISO recommendation [37]. Cell exposure was conducted using the undiluted extract and serial dilutions of 1:4, 1:8, 1:16, 1:32, and 1:64, with 100 µL applied to each well for 24 h. Culture medium without extract served as the negative control. Cell viability was quantified using the Cell Counting Kit-8 (CCK-8; Dojindo Laboratories, Kumamoto, Japan). After the 24 h exposure period, 10 µL of CCK-8 reagent was added to each well, and the plate was incubated for an additional 2 h at 37 °C with 5% CO_2_. Absorbance at 450 nm, reflecting live cell dehydrogenase activity, was measured using a SpectraMax M5 plate reader (Molecular Devices, Sunnyvale, CA, USA), and cell viability was expressed as a percentage relative to the negative control.

### 2.5. Statistical Analysis

The assumptions of normality and homogeneity of variance were verified using the Shapiro–Wilk test and Levene’s test, respectively. Statistical analysis was carried out using one-way ANOVA, followed by Tukey’s post hoc test for pairwise comparisons between groups. For the cytotoxicity assay, which involved both material group and extract dilution as variables, a separate one-way ANOVA was performed within each dilution level to evaluate differences among material groups at each dilution concentration. All data are presented as mean ± standard deviation (SD). All statistical analyses were conducted using SigmaPlot software version 12.0 (SYSTAT Software Inc., San Jose, CA, USA). The alpha level (α) was set at 5%.

## 3. Results

### 3.1. Flexural Strength and Elastic Modulus

**Figure 1:**
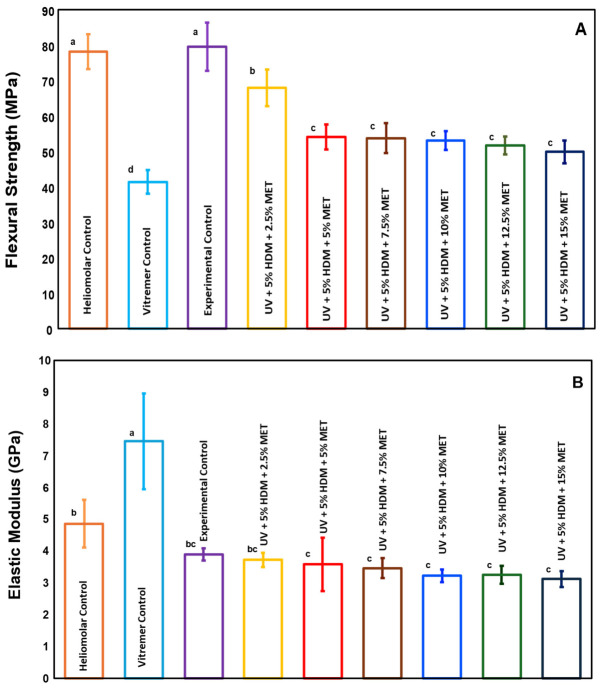
Mechanical characteristics for Heliomolar, Vitremer, experimental control, and experimental metformin groups are as follows: (**A**) Flexural Strength and (**B**) Elastic Modulus (mean ± SD; *n* = 6). Statistically significant (*p* < 0.05) values are represented using different letters across the groups.

The flexural strength of the experimental resins is shown in Figure 1A (mean ± SD; *n* = 6). The experimental control group had a flexural strength of 79.5 ± 6.8 MPa, showing no significant difference compared to the Heliomolar control at 78.1 ± 4.9 MPa (*p* > 0.1). As metformin concentration increased from 2.5% to 15%, flexural strength decreased in a dose-dependent manner, ranging from 67.9 ± 5.1 MPa to 50.1 ± 3.2 MPa for the UV + 5% HDM + 2.5% to 15% MET. However, all experimental groups (UV + 5% HDM + 2.5% to 15% MET) demonstrated flexural strengths significantly greater than the Vitremer control (41.3 ± 3.4) MPa (*p* < 0.05), while being significantly lower than that of the Heliomolar control (*p* < 0.05).

The elastic modulus of the different groups is shown in Figure 1B (mean ± SD; *n* = 6). Statistical analysis confirmed significant differences among all groups (p < 0.05). Vitremer control exhibited the highest elastic modulus of 7.4 ± 1.5 GPa, significantly higher than all other groups (p < 0.05). Heliomolar (4.8 ± 0.7) GPa showed a significantly higher elastic modulus than all experimental groups (p < 0.05), while being significantly lower than Vitremer (*p* < 0.05). The experimental control group and UV + 5% HDM + 2.5% to 15% MET groups demonstrated lower elastic moduli ranging from (3.8 ± 0.1) GPa to (3.1 ± 0.2) GPa, with no statistically significant differences observed among the UV + 5% HDM + 5% to 15% MET groups (p > 0.1).

### 3.2. Degree of Polymerization Conversion (DC)

**Figure 2:**
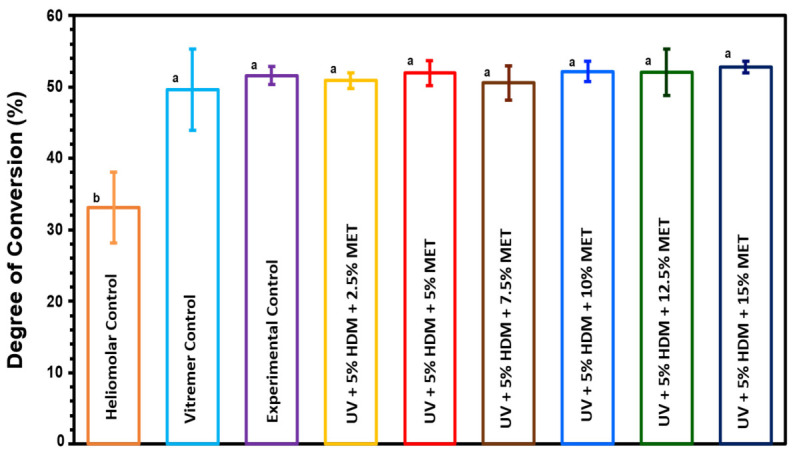
Comparison of the degree of polymerization conversion across Heliomolar, Vitremer, experimental control, and experimental metformin groups (*n* = 3; mean ± SD). Statistically significant (*p* < 0.05) values are represented using different letters across the groups.

The DC results for the different groups are summarized in Figure 2 (mean±SD; n=3). All experimental UV + 5% HDM + 2.5% to 15% MET groups exhibited DC values ranging from 50.2±2.1% to 53.4±1.8%, showing no significant difference between the different metformin concentrations (*p* > 0.1). However, a significant difference was observed compared to Heliomolar control (33.1±4.9)% (*p* < 0.05), while no significant variation was found when compared to Vitremer control (49.6±5.7)% (*p* > 0.1).

### 3.3. Composite Hardness

**Figure 3:**
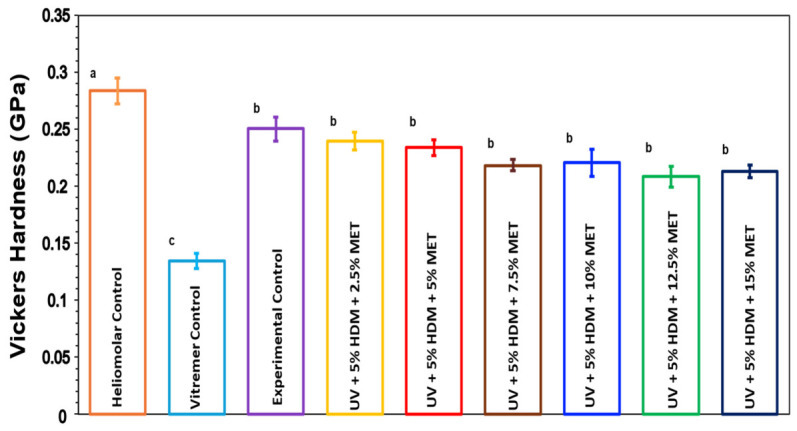
Vickers hardness for Heliomolar, Vitremer, experimental control, and experimental metformin groups. Results are shown as mean ± standard deviation (*n* = 5). Statistically significant (*p* < 0.05) values are represented using different letters across the groups.

Surface microhardness values are shown in Figure 3 (mean±SD; n=5). Heliomolar control showed the highest hardness at (0.28±0.01) GPa, which was significantly different from all other groups (*p* < 0.05). Within the experimental control and UV + 5% HDM + 2.5% groups reached (0.25±0.01) GPa and (0.23±0.01) GPa, respectively, while a significant concentration-dependent decrease (*p* < 0.05) was observed as metformin loading increased toward the UV + 5% HDM + 15% MET group. Notably, all experimental groups showed a significant difference compared to Vitremer control (0.13±0.006) GPa (*p* < 0.05), which exhibited the lowest microhardness.

### 3.4. Colony-Forming Unit (CFU/mL) Counts

**Figure 4:**
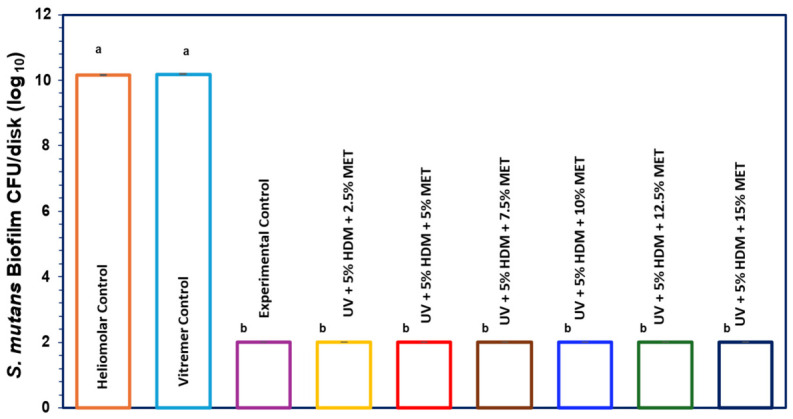
CFUs of *S. mutans* biofilm per disk (log10) for Heliomolar, Vitremer, experimental control, and experimental metformin groups. Data are presented as mean ± SD (*n* = 6). Statistically significant (*p* < 0.05) values are represented using different letters across the groups.

The CFU counts of 48 h *S. mutans* biofilms on the resin disks are shown in Figure 4 (mean±SD; n=6). Heliomolar and Vitremer control groups supported high bacterial growth at (3.2±0.5) × $10^{10}$ CFU/mL and (2.8±0.8) × $10^{10}$ CFU/mL, respectively, showing no significant difference between them (*p* > 0.1). The experimental control and UV + 5% HDM + 2.5% to 15% MET groups showed a significant reduction (*p* < 0.05) compared to both commercial controls. The inclusion of 5% DMAHDM in the resin resulted in an approximately 8-log reduction in CFU when compared to the two commercial groups. However, there were no statistically significant differences observed between the experimental control and metformin-loaded groups (*p* > 0.1).

### 3.5. Biofilm Metabolic Activity (MTT)

**Figure 5:**
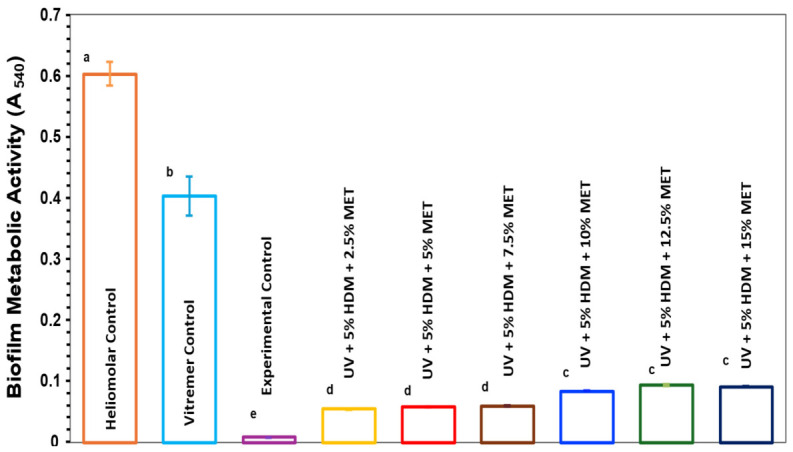
MTT assay evaluation of biofilm metabolic activity for Heliomolar, Vitremer, experimental control, and experimental metformin groups (mean ± SD; *n* = 6). The incorporation of 5% DMAHDM resulted in a 96% reduction in *S. mutans* metabolic activity. Statistically significant (*p* < 0.05) values are represented using different letters across the groups.

Figure 5 displays the metabolic activity of *S. mutans* biofilm on resin disks, as determined by the MTT assay after 48 h (mean ± SD; *n* = 6). The addition of DMAHDM to the resin resulted in a significant decrease in metabolic activity, ranging from approximately 84% to 91%, when compared to the Heliomolar control (0.603 ± 0.019) (*p* < 0.05). Specifically, experimental UV + 5% HDM + 2.5% to 15% MET groups exhibited values between (0.054 ± 0.001) and (0.093 ± 0.001), representing a substantial reduction in activity compared to both commercial controls, Heliomolar and Vitremer (0.402 ± 0.032). The experimental control group showed the lowest metabolic activity at (0.007 ± 0.003). No statistically significant differences were observed among the UV + 5% HDM + 10%, 12.5%, and 15% MET groups, which showed a slight plateau in metabolic activity (*p* > 0.1).

### 3.6. L-Lactic Acid Production

**Figure 6:**
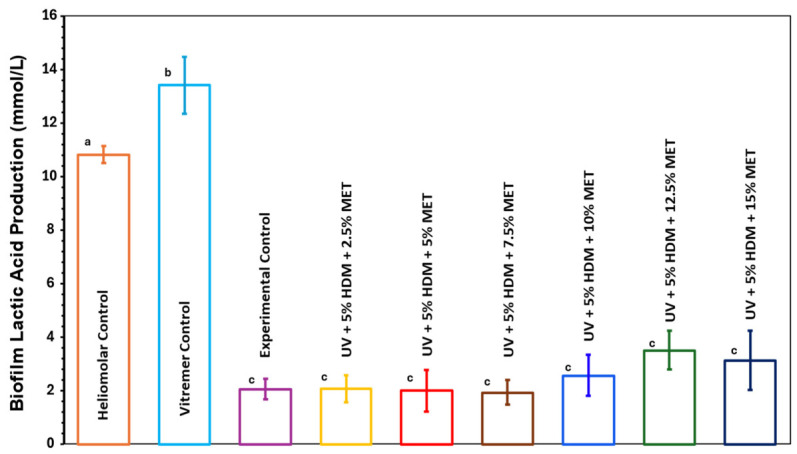
Lactic acid production by *S. mutans* biofilms in Heliomolar, Vitremer, experimental control, and experimental metformin groups. Values are presented as mean ± SD (*n* = 6). Statistically significant (*p* < 0.05) values are represented using different letters across the groups.

Figure 6 shows the L-lactic acid production of *S. mutans* biofilms on the resin disks (mean ± SD; *n* = 6). The experimental UV + 5% HDM + 2.5% to 15% MET groups exhibited a significant reduction in acid production by approximately 85% compared to the commercial controls (*p* < 0.05). The mean lactic acid concentration in the experimental groups showed a significant reduction of (1.9 ± 0.4) mmol/L, compared to Vitremer and Heliomolar controls, which exhibited the highest acid concentrations at (13.4 ± 1.6) mmol/L and (10.8 ± 0.3) mmol/L.

### 3.7. Cytotoxicity

**Figure 7:**
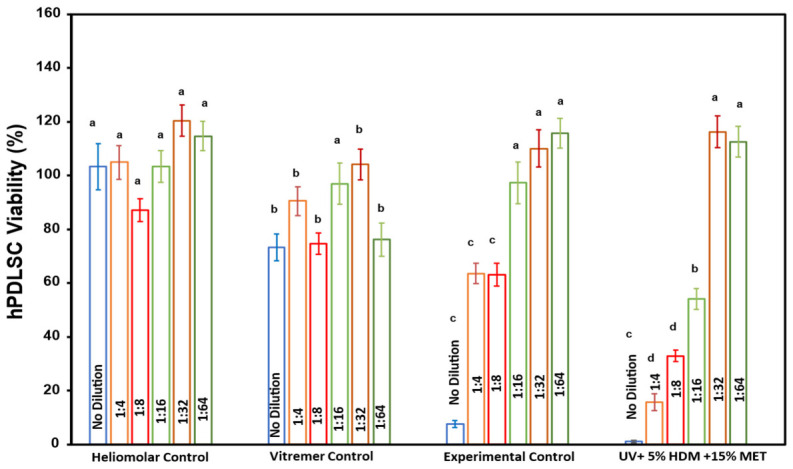
Cell viability of hPDLSCs exposed to the novel filling composite (mean ± SD; *n* = 3 × 3). Cell viability is provided for the experimental (UV + 5% HDM + 15% MET), commercial, and experimental control groups across several dilutions. The extract medium was serially diluted with fresh fibroblast medium at ratios of 1:4, 1:8, 1:16, 1:32, and 1:64, corresponding to extract/fresh medium volumes of 25/75 µL, 12.5/87.5 µL, 5.9/94.1 µL, 3.03/96.9 µL, and 1.5/98.4 µL, respectively. The 1:32 dilution simulates low salivary flow, while the 1:64 dilution simulates normal salivary flow. Different letters indicate statistically significant differences between the groups (*p* < 0.05).

Figure 7 presents the viability of hPDLSCs in response to a newly developed metformin-containing antibacterial dental resin material (UV + 5% HDM + 15% MET), in comparison to experimental control and commercial control groups (Heliomolar and Vitremer) (mean ± SD; *n* = 3 × 3). The x-axis shows dilutions for each group. Results show that when the extracts were introduced in their undiluted form, there was significant toxicity for the experimental control and for the UV + 5% HDM + 15% MET groups compared to the Heliomolar and Vitremer controls (*p* < 0.05). However, the experimental group containing 15% metformin at the 1:32 dilution had a notable decrease in toxicity, demonstrating acceptable cytocompatibility, with cell viability surpassing 75%.

## 4. Discussion

This study developed and characterized a novel metformin-containing antibacterial dental resin composite designed for root cavity restorations, specifically intended for non-stress-bearing areas of the dentition, where root surfaces are exposed due to periodontal attachment loss and gingival recession. The resin composite addresses two critical clinical objectives. These include the suppression of recurrent decay through DMAHDM antibacterial action and the potential stimulation of periodontal tissue regeneration via metformin incorporated as a bioactive filler within the composite matrix. Our findings indicate that these experimental composites possess mechanical properties suitable for non-load-bearing root cavity restorations, alongside potent contact-killing antibacterial activity against *S. mutans* and acceptable cytocompatibility toward hPDLSCs under diluted extract conditions, collectively supporting the therapeutic future potential of this novel material.

In evaluating these composites, it is vital to align their mechanical behavior with their specific clinical use-case: root surface restorations in areas characterized by periodontal recession. While posterior occlusal fillings must endure masticatory loads ranging from 200 to 800 N, cervical and non-bearing root restorations face significantly reduced mechanical pressure. While ISO 4049 mandates an 80 MPa flexural strength minimum for load-bearing polymers, this threshold is less stringent for cervical applications, where performance is traditionally benchmarked against glass ionomer cements [38]. The choice of using the UV resin system as the matrix for this composite was planned in consideration of many benefits of use compared to the traditional Bis-GMA/TEGDMA system utilized in most commercial dental composite products. Biocompatibility issues related to Bis-GMA are due to the fact that it is manufactured from Bisphenol-A (BPA), which is an endocrine disruptor. Both Bis-GMA and the hydrolysis byproduct of BPA are shown to leach out of cured composites and enter the saliva and/or adjacent soft tissues, which presents a significant concern regarding biocompatibility, especially since the material being used is expected to come into contact with the viable periodontal ligament stem cells for regenerative purposes [29,39]. In contrast, UDMA lacks a BPA core and is therefore inherently more biocompatible, making it the preferred base monomer for applications requiring close proximity to viable tissue [40]. Furthermore, Bis-GMA exhibits extremely high viscosity (approximately 500–800 Pa·s) and therefore requires the addition of a reactive diluent, typically TEGDMA, to achieve workable consistency. TEGDMA, however, is a documented cytotoxin and genotoxin, and its incorporation introduces an additional source of biological risk through monomer leaching [40]. The UV resin system circumvents this requirement: UDMA possesses inherently lower viscosity due to its flexible aliphatic urethane backbone, and TEG-DVBE, an ether-based divinyl monomer, serves as a low-shrinkage stress co-monomer that further reduces polymerization shrinkage stress without the cytotoxic profile of TEGDMA [33]. Prior studies have demonstrated that UDMA/TEG-DVBE composites exhibit significantly lower polymerization shrinkage stress than Bis-GMA/TEGDMA composites while maintaining equivalent or superior mechanical performance [41,42]. This reduction in shrinkage stress is of particular clinical relevance in root cavity restorations, where the thin cervical dentin walls are highly susceptible to stress-induced marginal gap formation and microleakage, primary drivers of recurrent root caries. In this study, the experimental groups met these clinical standards. Notably, even the experimental UV + 5% HDM + 15% MET resin group (50.1 ± 3.2) MPa, despite falling just below the posterior ISO limit, remains a viable clinical candidate for root restorations where resisting low-magnitude cervical stress is the primary requirement.

The flexural strength data indicated a trend toward reduced flexural strength as the concentration of metformin increased. The values ranged from an average of 79.5 ± 6.8 MPa in the experimental controls to 50.1 ± 3.2 MPa in the UV + 5% HDM + 15% MET group. Bhadila et al. demonstrated that composites incorporating bioactive agents as co-fillers, which is analogous to the role of metformin in the present study, can maintain clinically acceptable flexural strength at low-to-moderate loading concentrations, with degradation observed only at higher inclusion levels [29]. Vitremer commercial control is widely used for root caries restoration. It had a lower flexural strength of 41.3 ± 3.4 MPa, which is below the ISO 4049 minimum threshold [38]. In contrast, all experimental composites demonstrated flexural strengths exceeding the current clinical benchmark for root cavity restoration, supporting their suitability for root caries restoration applications. The experimental control group demonstrated a flexural strength value comparable to Heliomolar (*p* > 0.1); whereas each of the experimental groups containing metformin was found to have significantly lower flexural strength than Heliomolar (*p* < 0.05). These differences reflect a progressive mechanical impact of metformin incorporation. However, all experimental groups UV + 5% HDM + 2.5 to 15% MET exhibited flexural strengths meeting or exceeding the ISO 4049 minimum flexural strength threshold of 50 MPa, confirming their clinical acceptability for root cavity restoration across all tested metformin concentrations.

The elastic modulus values of experimental UV + 5% HDM + 2.5% to 15% MET groups ranged from 3.7 to 3.1 GPa, which were significantly lower than Vitremer (7.4 ± 1.5) GPa (*p* < 0.05). The elastic moduli at the cervical, middle, and apical third of the buccal surface of a tooth are 4.4 ± 2.4 GPa, 3.4 ± 2.0 GPa, and 2.4 ± 1.8 GPa, respectively, which gradually decrease from the cervical toward the apical third of the root [43]. The elastic modulus values of the experimental composites are therefore within a comparable range to the surrounding cementum substrate, which may help reduce interfacial stress concentration at the restoration margin. ISO 4049 [38] does not specify a minimum elastic modulus for non-load-bearing restorations. In fact, a lower elastic modulus may be mechanically advantageous in root surface applications, where the surrounding cervical dentin is thinner and more susceptible to stress concentration. Prior studies on cervical composite restorations have suggested that a closer modulus match between the restorative material and tooth structure reduces interfacial stress and the risk of cohesive or adhesive failure [2]. The intermediate modulus values of the experimental composites thus may represent a more clinically appropriate mechanical flexibility for non-load-bearing root surface restorations compared to the notably stiffer Vitremer. No statistically significant variation in elastic modulus was observed among experimental UV + 5% HDM + 5% to 15% MET groups (*p* > 0.1), confirming that varying metformin content within this range does not further alter structural stiffness.

The DC of all experimental groups ranged from 50.2 ± 2.1% to 53.4 ± 1.8%, values significantly higher than Heliomolar (33.1 ± 4.9)% (*p* < 0.05) and comparable to Vitremer (49.6 ± 5.7)% (*p* ≥ 0.05). ISO 4049 [38] does not specify a mandatory minimum DC; however, a higher DC is clinically desirable as it reflects more complete polymerization of the resin matrix, reducing residual monomer content and minimizing potential cytotoxic effects on surrounding tissues. Wang et al. demonstrated that UV-based resins achieve superior photopolymerization efficiency compared to conventional Bis-GMA systems, attributable to the optimized 1:1 molar ratio and complementary polymerization kinetics of this monomer system [23], a finding directly corroborated by the high DC values obtained in the present study. Consistent DC values across all metformin concentration groups confirm that the addition of metformin as a co-filler does not interfere with the photopolymerization process.

Surface microhardness showed a significant concentration-dependent decrease with increasing metformin loading (*p* < 0.05), consistent with the progressive replacement of hard barium glass fillers (which provide mechanical reinforcement) with softer metformin particles. Nevertheless, all experimental groups demonstrated hardness values significantly higher than Vitremer (0.13 ± 0.006) GPa (*p* < 0.05), which is the current clinical standard for root caries restoration. Bhadila et al. similarly reported concentration-dependent hardness reductions in composites containing bioactive co-fillers, while maintaining values above clinically accepted benchmarks [29]. In the context of non-load-bearing root restorations, where the primary hardness requirement is resistance to toothbrushing wear and soft tissue contact rather than occlusal abrasion, the hardness values across all experimental groups are well-suited to the clinical environment. Taken together, the full mechanical dataset, flexural strength meeting and exceeding ISO 4049 thresholds across nearly all experimental groups, clinically appropriate elastic modulus, superior DC compared to Heliomolar, and hardness consistently above the Vitremer benchmark strongly support the suitability of the experimental composites for use in non-load-bearing root cavity restorations.

All DMAHDM-containing experimental composites demonstrated potent antibacterial activity against *S. mutans* biofilms, achieving an approximately 8-log reduction in CFU counts compared to commercial controls. This near-total elimination of viable bacteria on the resin surface is consistent with the well-established contact-killing antibacterial mechanism of DMAHDM, in which the long alkyl chain of the quaternary ammonium monomer penetrates and disrupts bacterial cell membranes upon direct contact, without releasing a diffusible antibacterial agent. This intrinsic mechanism confers durable antibacterial activity that is not dependent on drug reservoir depletion, overcoming a fundamental limitation of chlorhexidine-releasing composites, which lose efficacy as the drug is exhausted over time [8]. Oliveira et al. comprehensively reviewed the antibacterial properties of DMAHDM-containing composites, confirming consistent 4–8 log reductions in *S. mutans* CFUs across multiple studies [11], directly in line with the 8-log reduction observed here. Zhang et al. further demonstrated that DMAHDM-containing composites retain long-term antibacterial activity against *S. mutans* without significant reduction in mechanical performance over time [12], supporting the durability of the antibacterial function in the present composite.

The MTT assay revealed reductions in *S. mutans* biofilm metabolic activity of 84–91% in DMAHDM-containing experimental groups compared to Heliomolar (*p* < 0.05). Suppression of metabolic activity at this magnitude indicates broad inhibition of bacterial energy production and vital cellular processes essential for biofilm maturation and virulence. This finding aligns with the work of Zhou et al., who demonstrated that DMAHDM-containing composites significantly suppressed *S. mutans* metabolic activity at margins of enamel restorations and reduced secondary caries formation in vitro [19].

Lactic acid production was reduced by approximately 85% in all experimental groups compared to commercial controls (*p* < 0.05), with mean lactic acid concentrations falling to (1.9 ± 0.4) mmol/L versus (13.4 ± 1.6) mmol/L for Vitremer and (10.8 ± 0.3) mmol/L for Heliomolar. Lactic acid is the primary mediator of cariogenic demineralization of root cementum and dentin, and its near elimination is of direct clinical significance for the prevention of recurrent root caries at restoration margins [1,2]. Clarin et al. similarly reported substantial reductions in lactic acid production in DMAHDM nanocomposites, confirming the functional antibacterial efficacy of this class of materials [13].

Critically, no statistically significant variation in lactic acid production, CFU counts, or metabolic activity was detected across the different metformin concentration groups (*p* > 0.1), confirming that DMAHDM, not metformin, is the principal antibacterial agent in the composite. The addition of metformin at any tested concentration neither enhances nor diminishes the antibacterial performance of the material. This functional separation of roles, contact-killing antibacterial activity from DMAHDM and local delivery of a regenerative agent from metformin, represents a well-designed dual-function therapeutic strategy that maximizes clinical utility without mechanical or biological compromise between the two components.

The cytotoxicity assessment was conducted using eluates from the UV + 5% HDM + 15% MET group alongside the experimental control and commercial controls (Heliomolar and Vitremer). The experimental UV + 5% HDM + 15% MET group, the highest metformin concentration tested, was chosen as the representative experimental group for cytotoxicity evaluation. Across all antibacterial outcomes measured (CFU reduction, metabolic activity suppression, and lactic acid reduction), the UV + 5% HDM + 15% MET group showed no statistically significant difference from any lower-concentration experimental group (*p* > 0.1), demonstrating equivalent antibacterial efficacy regardless of metformin content. Furthermore, the UV + 5% HDM + 15% MET group maintained clinically acceptable mechanical properties for a non-load-bearing root restoration, having a flexural strength of 50.1 MPa, a degree of conversion consistent with other experimental groups, and surface hardness significantly above that of Vitremer. Given that the UV + 5% HDM + 15% MET group carries the highest metformin payload and therefore offers the greatest potential for local regenerative bioactive function, and since its mechanical and antibacterial performance did not significantly diverge from lower-concentration groups, it was selected as the most informative and conservative group for biological safety assessment. This rationale follows the standard principle in biomaterials testing of evaluating the most extreme formulation: demonstrating acceptable cytocompatibility at the highest drug loading provides a safety envelope that logically extends to all lower concentrations.

The cytotoxicity results showed that undiluted eluates from both the experimental control and UV + 5% HDM + 15% MET groups exhibited significant toxicity toward hPDLSCs compared to commercial controls (*p* < 0.05). This initial toxicity at full-strength extract concentration is a well-documented and expected phenomenon in dental composite research, primarily attributable to the leaching of residual unreacted monomers, particularly TEG-DVBE and UDMA, from freshly prepared specimens [7]. It is important to note that ISO 10993-5 defines cytotoxicity as cell viability falling below 70% of the negative control [37]. At a 1:32 dilution, the UV + 5% HDM + 15% MET group achieved cell viability exceeding 75%, meeting the ISO 10993-5 threshold for acceptable cytocompatibility. This dilution level closely approximates the in vivo conditions surrounding a placed restoration, where residual monomer eluates are progressively diluted by salivary flow, tissue fluids, and diffusion barriers within the pulpal and periodontal tissues. It has been reported that initial monomer leaching from resin composites produces cytotoxic effects in vitro at undiluted concentrations, which diminish substantially at physiologically representative dilutions [44], consistent with the dose-dependent pattern observed in the present study. The commercial controls showed higher cell viability at undiluted concentrations, reflecting their distinct and more extensively aged monomer compositions; however, neither Heliomolar nor Vitremer possesses any potential regenerative bioactive function, which is the defining therapeutic advantage of the experimental composite.

The clinical validity of these dilution levels is directly supported by salivary physiology. Unstimulated (resting) salivary flow, representing conditions such as sleep, hyposalivation, or medically compromised elderly patients who are also at the highest risk for root caries, averages approximately 0.1–0.3 mL/min, and in this low-flow state, a dilution factor in the range of 1:32 conservatively approximates the dilution of composite eluates by the oral fluid environment. Normal unstimulated flow (0.3–0.5 mL/min) and stimulated salivary flow rates exceeding 1.0–2.0 mL/min during eating or chewing produce substantially greater dilution of eluates at the restoration surface, corresponding more closely to the 1:64 dilution tested in the present study. This is consistent with the observation that in vitro extract-based cytotoxicity testing inherently overestimates clinical tissue exposure because the dynamic oral environment, including continuous salivary flow, buffering capacity, and enzymatic clearance, continuously dilutes and clears material eluates from the tissue interface [45]. The fact that the UV + 5% HDM + 15% MET group achieved cell viability exceeding the ISO 10993-5 threshold of 70% already at the conservative 1:32 dilution simulating the worst-case scenario of low salivary flow in hyposalivation patients indicates that acceptable cytocompatibility would be expected across the full spectrum of salivary flow conditions encountered clinically, with the 1:64 dilution providing an additional margin of safety for patients with normal to high salivary secretion rates. However, it should be acknowledged that the correlation between in vitro dilution factors and actual intraoral eluate concentrations has not been experimentally validated in this study, and these dilution approximations should therefore be interpreted with appropriate caution.

While the present study did not assess osteogenic differentiation endpoints, including alkaline phosphatase activity and mineral deposition, these represent critical next steps in the biological characterization of this composite. Prior in vitro studies have demonstrated that metformin stimulates the proliferation and osteogenic differentiation of hPDLSCs through AMPK signaling activation [16,20], and a recent study confirmed that metformin delivered from a dental resin matrix promotes both osteogenic and cementogenic differentiation of hPDLSCs [22]. The favorable cytocompatibility demonstrated in the present study establishes the biological safety foundation upon which future investigations of the osteogenic and periodontal regenerative potential of this composite can be built.

The present study advances a growing body of evidence supporting the integration of metformin into dental biomaterials for regenerative applications. Yu et al. reported that a metformin-containing dental resin stimulated osteogenic and cementogenic differentiation of hPDLSCs in vitro and promoted periodontal and tooth root regeneration [22]. Qiao et al. demonstrated that the combination of hPDLSCs and metformin via organic cation transporters significantly enhanced periodontal tissue regeneration in a rat model [21]. The present study extends this work by incorporating metformin into an antibacterial composite matrix alongside DMAHDM, producing a dual-function material that combines the regenerative properties of metformin, as demonstrated in prior studies [16,20,22], with the potent contact-killing antibacterial activity of DMAHDM in a single restorative platform, an approach not previously reported in the literature.

In comparison to prior DMAHDM-containing composites developed without a bioactive regenerative component, the addition of metformin introduces a therapeutic dimension that transforms the material from a passive caries-preventing restoration into a potential local promoter of periodontal tissue repair. Makvandi et al. systematically reviewed quaternary ammonium compounds in dental materials, concluding that contact-killing antibacterial monomers such as DMAHDM represent the most durable and promising class of antibacterial dental restoratives [10]. The present study confirms and builds upon these conclusions while demonstrating that the simultaneous incorporation of a bioactive molecule does not compromise this antibacterial performance. The antibacterial efficacy demonstrated here also substantially surpasses that of Vitremer, the current glass ionomer standard for root caries management, across all three biofilm endpoints evaluated: CFU counts, metabolic activity, and lactic acid production.

Several biological limitations need to be considered when evaluating the results of the current study. The in vitro nature of this study represents an inherent limitation, as laboratory conditions do not fully reproduce the multifactorial biological, mechanical, and microbial complexities encountered clinically in the oral cavity. The antibacterial experiments employed a single-species biofilm model using *S. mutans*, which, while the primary cariogenic pathogen in root caries, does not replicate the polymicrobial complexity of the oral biofilm environment in vivo. Future studies should assess metformin release from the resin disks, live/dead staining, alizarin red S staining of minerals synthesized by the cells, alkaline phosphatase activity, and quantitative real-time reverse transcription PCR.

## 5. Conclusions

The current study successfully designed a novel multifunctional composite for root cavity restoration. Metformin was incorporated with DMAHDM in a UDMA/TEG-DVBE resin matrix for the first time. The resulting material maintained adequate mechanical properties, a strong contact-killing antibacterial effect, and acceptable cytocompatibility. The experimental composites showed flexural strength up to 67.9 MPa, significantly exceeding the Vitremer control (41.3 MPa). The antibacterial testing showed an 8-log reduction in *S. mutans* CFUs and a 92% decrease in biofilm metabolic activity. The material also showed acceptable cytocompatibility toward hPDLSCs under diluted extract conditions, with cell viability exceeding 75% (*p* > 0.1). These results establish this material as a promising root cavity restorative with dual potential for preventing recurrent root caries and potentially promoting periodontal tissue regeneration.

## Figures and Tables

**Table 1:** Shows the final experimental and control groups.

Group Description	Abbreviation
Commercial Control 1	Heliomolar control
Commercial Control 2	Vitremer control
UV + 5% DMAHDM + 70% Glass fillers + 0% metformin	Experimental control
UV + 5% DMAHDM + 67.5% Glass fillers + 2.5% metformin	UV + 5% HDM + 2.5% MET
UV + 5% DMAHDM + 65% Glass fillers + 5% metformin	UV + 5% HDM + 5% MET
UV + 5% DMAHDM + 62.5% Glass fillers + 7.5% metformin	UV + 5% HDM + 7.5% MET
UV + 5% DMAHDM + 60% Glass fillers + 10% metformin	UV + 5% HDM + 10% MET
UV + 5% DMAHDM + 57.5% Glass fillers + 12.5% metformin	UV + 5% HDM + 12.5% MET
UV + 5% DMAHDM + 55% Glass fillers + 15% metformin	UV + 5% HDM + 15% MET

## Data Availability

The original contributions presented in this study are included in the article. Further inquiries can be directed to the corresponding authors.

## Abbreviations

The following abbreviations are used in this manuscript:

DMAHDM	Dimethylaminohexadecyl methacrylate
TEG-DVBE	Triethylene glycol divinylbenzyl ether
UDMA	Urethane dimethacrylate,
hPDLSCs	Human Periodontal Ligament Stem Cells
UV	UDMA + TEG-DVBE
